# Synapsin I and Synapsin II regulate neurogenesis in the dentate gyrus of adult mice

**DOI:** 10.18632/oncotarget.24655

**Published:** 2018-04-10

**Authors:** Raffaella Barbieri, Andrea Contestabile, Maria Grazia Ciardo, Nicola Forte, Antonella Marte, Pietro Baldelli, Fabio Benfenati, Franco Onofri

**Affiliations:** ^1^ Department of Experimental Medicine, University of Genova, 16132, Genova, Italy; ^2^ Department of Neuroscience and Brain Technologies, Istituto Italiano di Tecnologia, 16163, Genova, Italy; ^3^ Center for Synaptic Neuroscience and Technology, Istituto Italiano di Tecnologia, 16132, Genova, Italy

**Keywords:** adult neurogenesis, dentate gyrus, synapsins, brain-derived neurotrophic factor, epilepsy

## Abstract

Adult neurogenesis is emerging as an important player in brain functions and homeostasis, while impaired or altered adult neurogenesis has been associated with a number of neuropsychiatric diseases, such as depression and epilepsy. Here we investigated the possibility that synapsins (Syns) I and II, beyond their known functions in developing and mature neurons, also play a role in adult neurogenesis. We performed a systematic evaluation of the distinct stages of neurogenesis in the hippocampal dentate gyrus of Syn I and Syn II knockout (KO) mice, before (2-months-old) and after (6-months-old) the appearance of the epileptic phenotype. We found that Syns I and II play an important role in the regulation of adult neurogenesis. In juvenile mice, Syn II deletion was associated with a specific decrease in the proliferation of neuronal progenitors, whereas Syn I deletion impaired the survival of newborn neurons. These defects were reverted after the appearance of the epileptic phenotype, with Syn I KO and Syn II KO mice exhibiting significant increases in survival and proliferation, respectively. Interestingly, long-term potentiation dependent on newborn neurons was present in both juvenile Syn mutants while, at later ages, it was only preserved in Syn II KO mice that also displayed an increased expression of brain-derived neurotrophic factor. This study suggests that Syns I and II play a role in adult neurogenesis and the defects in neurogenesis associated with Syn deletion may contribute to the alterations of cognitive functions observed in Syn-deficient mice.

## INTRODUCTION

Adult neurogenesis is a structural form of neural plasticity that contributes to the maintenance of normal brain function and homeostasis [1]. Specifically, within the hippocampal dentate gyrus (DG), newborn dentate granule cells are known to integrate into the pre-existing circuitry and play a substantial role in certain types of learning and memory and in the modulation of emotional behavior or anxiety [2, 3]. Almost every stage of this process, from proliferation to differentiation, survival and integration of new neurons, can be critically modulated by a number of environmental and physiological factors, including diet, exercise and aging, or pathological conditions like epilepsy or neurodegenerative disorders [4–8]. At the molecular level, each step involves complex interactions among cytokines, neurotrophins and presynaptic proteins [9].

Among presynaptic proteins the Synapsins (Syns), an abundant family of neuron-specific synaptic vesicle (SV)-associated phosphoproteins, play important roles in both neuronal development and synaptic physiology [10, 11]. Syns represent a convergence point for distinct signal transduction pathways, since they are substrates for cAMP-dependent, Ca^2+^/calmodulin-dependent I and II, mitogen-activated protein kinases, cyclin-dependent kinase-5 and pp60^c-src^. In mammals Syns are encoded by three distinct genes (*SynI*, *SynII* and *SynIII*) that generate more than ten isoforms by differential splicing. These transcripts are composed of a mosaic of individual and shared domains that are highly conserved during evolution, and their expression is developmentally regulated (see [11] for review).

Nonsense and missense mutations in *SYN1/SYN2* genes have been identified as causative for epilepsy and autism spectrum disorder (ASD) in humans [12–16]. In addition, multicenter gene mapping analysis identified *SYN2* as one of the main genes contributing to epilepsy predisposition [17]. Interestingly, genetically altered mice lacking Syn I, Syn II or both are all prone to partial, secondarily generalized epileptic seizures, which appear at 2-3 months of age, and progressively aggravate with age [18–21] and exhibit cognitive disturbances compatible with ASD [22–24]. The epileptic propensity of Syn knockout (KO) mice is associated with dysfunctions in the excitatory/inhibitory balance in the hippocampus, observed well before the emergence of the overt epileptic phenotype and consisting in a primary impairment of synchronous and asynchronous GABA release [25–30].

Syn III is the most precociously expressed Syn isoform, consistent with a role in neuronal development [31]. Indeed, Syn III is expressed in nestin-positive neural progenitors and its constitutive depletion affects adult neurogenesis in the DG of the hippocampus by decreasing cell proliferation and enhancing survival of neural progenitors [32]. Moreover, *in vivo* downregulation of Syn III expression caused defects in migration and orientation of layer II/III neocortical pyramidal neurons [33, 34].

As Syns I and II have been shown to participate in neuronal development *in vitro* (see [10] for review), we addressed the possibility that these isoforms, which are predominantly expressed by mature neurons [31, 35], are also involved in the regulation of adult neurogenesis. Thus, we performed a systematic evaluation of the distinct stages of neurogenesis in the hippocampal DG of wild type (WT), Syn I KO and Syn II KO mice, before (2-months-old) and after (6-months-old) the appearance of the epileptic phenotype.

We found that Syns I and II, notwithstanding their later expression profile with respect to Syn III, also play important roles in the regulation of adult neurogenesis. Before the appearance of epilepsy, Syn II deletion is associated with a specific decrease of the proliferation of neuronal progenitors, whereas Syn I deletion impairs the survival of the newborn neurons. By contrast, after the appearance of the overt epileptic phenotype, these defects are reverted and survival and proliferation result significantly increased in Syn I KO and Syn II KO mice, respectively. Similar to WT mice, juvenile Syn I KO and Syn II KO mice expressed newborn neuron-dependent long-term potentiation (LTP), while this form of LTP was preserved at later ages only in Syn II KO mice that also displayed an increased expression of brain-derived neurotrophic factor (BDNF). The data indicate that Syns I and II play a role in adult neurogenesis.

## RESULTS

### Synapsins I and II regulate proliferation and survival of neural progenitor cells in the adult dentate gyrus

**Figure 1:**
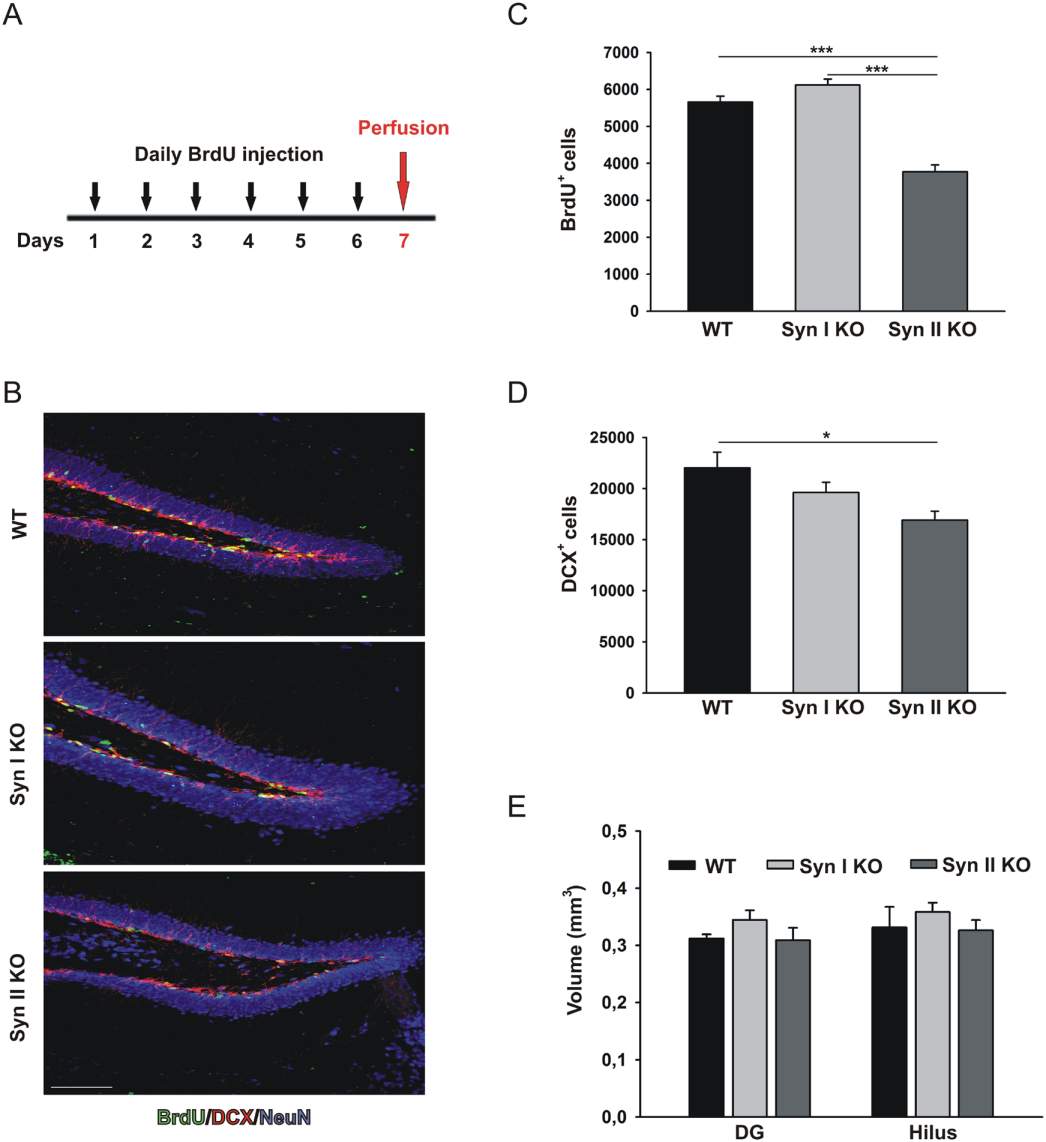
Neuronal progenitor proliferation in the DG of juvenile Syn I KO and Syn II KO mice **(A)** Experimental paradigm for BrdU injections to label proliferating cells. **(B)** Representative images of hippocampal sections from 2-months-old WT, Syn I KO and Syn II KO male mice stained for BrdU (green), DCX (red) and NeuN (blue). Scale bar, 100 μm. **(C, D)**. Number of BrdU-positive (C) and DCX-positive (D) cells in the subgranular zone of DG of WT (n=6), Syn I KO (n=4) and Syn II KO (n=6) mice. **(E)** Quantification of DG and hilus volumes (in mm^3^) in the three genotypes (n=5 for each genotype). Data are presented as means ± sem. ^*^p<0.05, ^***^p< 0.001, one-way ANOVA/Bonferroni’s tests.

In order to examine the proliferation of neural progenitor cells (NPCs) in the hippocampal DG of 2-months-old Syn I KO and Syn II KO mice, animals were treated daily with 5-bromo-2′-deoxyuridine (BrdU) injections for six consecutive days. One day after the last pulse, mice were perfused (Figure 1A) and hippocampal sections processed for BrdU staining. Compared with WT and Syn I KO mice, the number of BrdU-positive cells was significantly decreased in Syn II KO, indicating a marked reduction in hippocampal progenitor proliferation (Figure 1B, 1C). Similarly, the quantification of the total number of cells positive for doublecortin (DCX), a marker of neuroblasts and newly generated neurons, revealed a significant decrease in Syn II KO mice, suggesting that the impairment in proliferation produces a parallel decrease in the number of newborn neurons (Figure 1B, 1D). However, the reduction in adult hippocampal neurogenesis was not associated with any change in global DG or hilus volumes that were similar across genotypes (Figure 1E).

**Figure 2:**
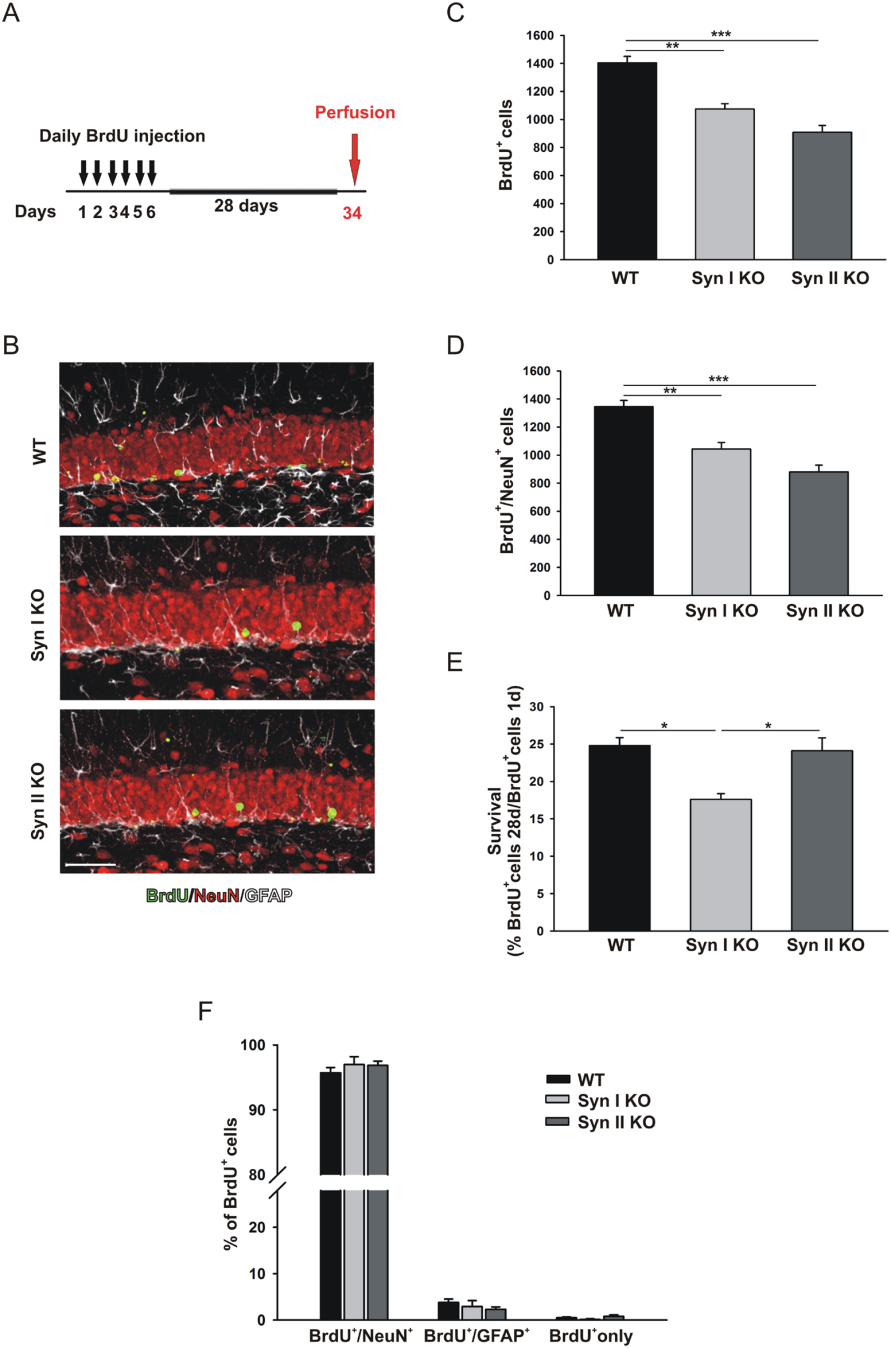
Survival and differentiation of neuronal progenitors in the DG of juvenile Syn I KO and Syn II KO mice **(A)** Experimental paradigm for BrdU injections to label surviving cells. **(B)** Representative images of hippocampal sections from 2-months-old WT, Syn I KO and Syn II KO male mice stained for BrdU (green), NeuN (red) and GFAP (white). Scale bar, 50 μm. **(C, D)** Number of BrdU-positive (C) and Brdu/NeuN double-positive (D) cells in the subgranular zone of DG of WT (n=6), Syn I KO (n=4) and Syn II KO (n=7) mice. **(E)** Survival rates calculated as percentages of BrdU-positive cells at 28 days with respect to those positive at 1 day after the last BrdU injection. **(F)** Percentages of BrdU-positive cells that had differentiated into neurons (BrdU/NeuN double-positive) or glia (BrdU/GFAP double-positive) or remained undifferentiated (BrdU/BrdU only). Data are presented as means ± sem. ^*^p<0.05, ^**^p<0.01, ^***^p< 0.001, One-way ANOVA/Bonferroni’s tests.

The survival of the progeny of dividing progenitor cells was determined through BrdU pulse-chase experiments, by counting the number of BrdU-positive cells and BrdU/NeuN double-positive cells found 28 days after the last BrdU administration (Figure 2A). The survival rate (*i.e.*, the percentage of BrdU-positive cells counted after 28 days over BrdU-positive cells found one day after the last injection) in WT mice was comparable to those observed in previous studies (see, e.g., [32]). Interestingly, a significant reduction of the number of BrdU-positive cells at 28 days was observed in both Syn I KO and Syn II KO mice with respect to WT mice (Figure 2B–2D). However, when the reduced proliferation found in Syn II KO mice was considered (Figure 1C), the survival rate resulted indistinguishable from that observed in WT animals (Figure 2E). By contrast, the percentage of survival of progenitor cells was significantly impaired in Syn I KO mice that were characterized by a normal proliferative activity (Figure 2E).

Differentiation of the surviving progenitors into neurons or glia was also examined by co-labeling of BrdU-positive cells with the mature neurons marker NeuN and the astrocyte marker glial fibrillary acidic protein (GFAP). The percentage of differentiated neuronal and glial cells or undifferentiated cells, calculated on the total amount of BrdU-positive cells, was similar across genotypes (Figure 2F), suggesting that the absence of either Syn I or Syn II did not alter the fate determination of dentate progenitors.

### The defects in adult neurogenesis of Synapsin I and Synapsin II KO mice are reverted during the symptomatic state of epilepsy

**Figure 3:**
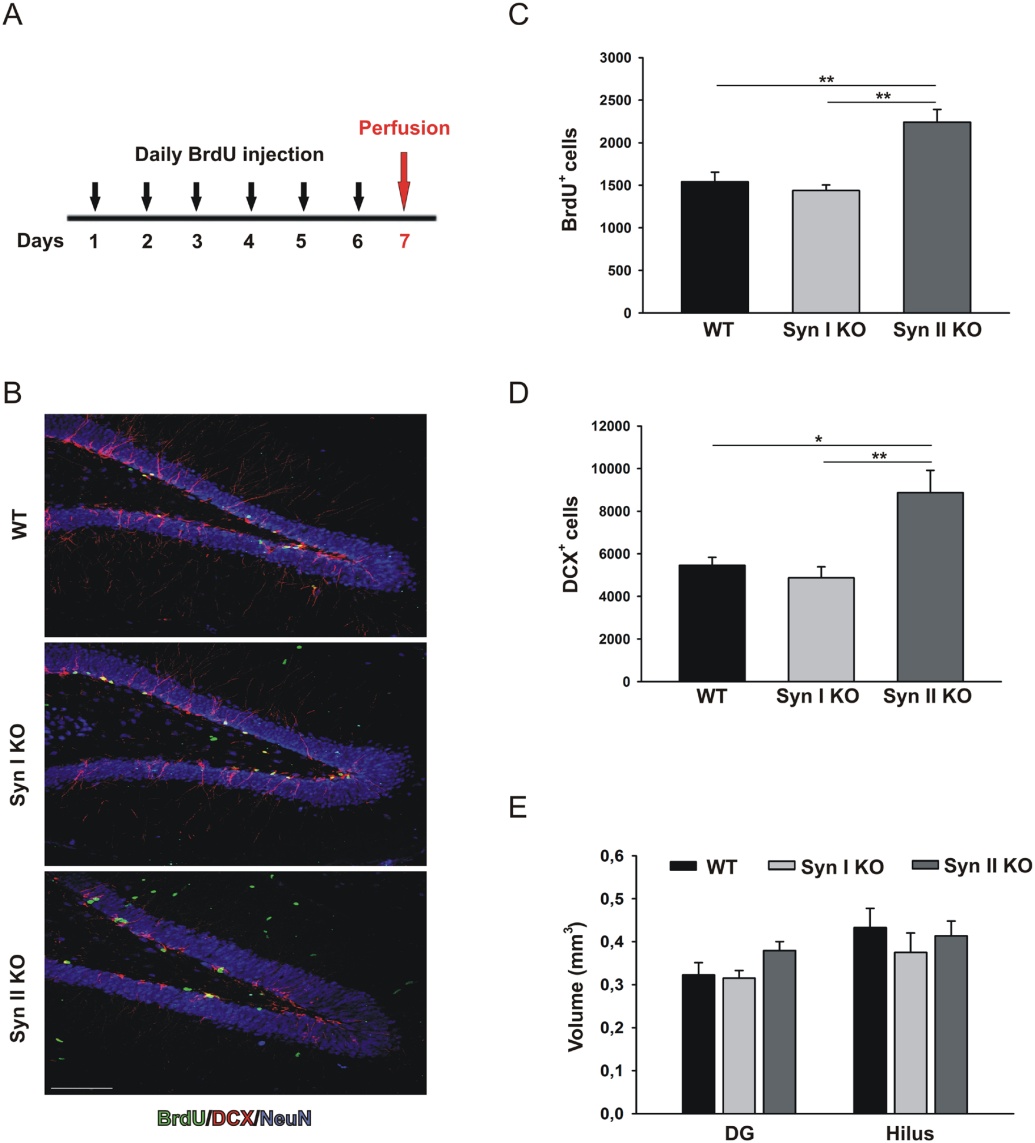
Neuronal progenitor proliferation in the DG of symptomatic Syn I KO and Syn II KO mice **(A)** Experimental paradigm for BrdU injections to label proliferating cells. **(B)** Representative images of hippocampal sections from 6-months-old WT, Syn I KO and Syn II KO male mice stained for BrdU (green), DCX (red) and NeuN (blue). Scale bar, 100 μm. **(C, D)** Number of BrdU-positive (C) and DCX-positive (D) cells in the subgranular zone of DG of WT, Syn I KO and Syn II KO mice (n=5 per genotype). **(E)** Quantification of DG and hilus volumes (in mm^3^) in the three genotypes (n=5 for each genotype). Data are presented as means ± sem. ^*^p<0.05, ^**^p<0.01, one-way ANOVA/Bonferroni’s tests.

**Figure 4:**
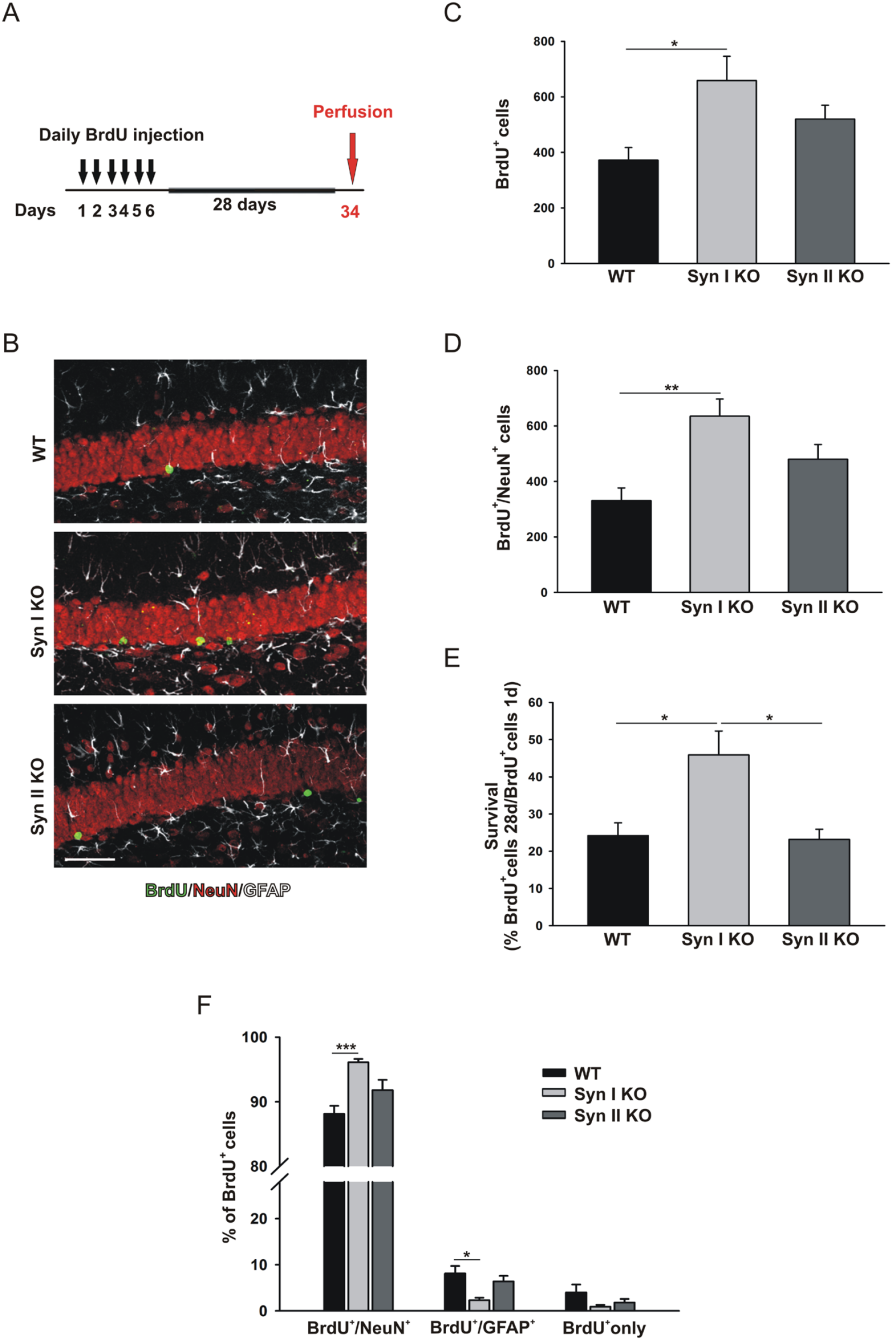
Survival and differentiation of neuronal progenitors in the DG of symptomatic Syn I KO and Syn II KO mice **(A)** Experimental paradigm for BrdU injections to label surviving cells. **(B)** Representative images of hippocampal sections from 6-months-old WT, Syn I KO and Syn II KO male mice stained for BrdU (green), NeuN (red) and GFAP (white). Scale bar, 50 μm. **(C, D)** Number of BrdU-positive (C) and Brdu/NeuN double-positive (D) cells in the subgranular zone of DG of WT (n=7), Syn I KO (n=6) and Syn II KO (n=4) mice. **(E)** Survival rates calculated as percentages of BrdU-positive cells at 28 days with respect to those positive at 1 day after the last BrdU injection. **(F)** Percentages of BrdU-positive cells that had differentiated into neurons (BrdU/NeuN double-positive) or glia (BrdU/GFAP double-positive) or remained undifferentiated (BrdU/BrdU only). Data are presented as means ± sem. ^*^p<0.05, ^**^p<0.01, ^***^p< 0.001, one-way ANOVA/Bonferroni’s tests.

We next evaluated proliferation, survival and differentiation rates in 6-months-old animals, when both Syn I KO and Syn II KO mice had developed an overt epileptic phenotype, by applying the same BrdU labeling paradigm used above and comparing the phenotype with that of age-matched WT littermates (Figure 3A; Figure 4A). Strikingly, the defects observed in young, non-epileptic Syn KO mice were reverted in epileptic mice of the same genotype. In fact, Syn II mice showed a significant increase in the proliferative activity of NPCs (BrdU-positive cells) that was paralleled by a higher abundance of newborn neurons (DCX-positive cells) with respect to either WT or Syn I KO mice (Figure 3B–3D). Also at this age, the volumes of the DG or the hilus in mutant mice were not significantly different from those of WT controls (Figure 3E).

By contrast, Syn I KO, but not Syn II KO, mice showed a significant increase in both total number of BrdU-labelled cells and total amount of BrdU/NeuN double-positive cells found 28 days after the last BrdU injection, when compared to WT mice (Figure 4B–4D). Accordingly, the survival rate of BrdU-labelled cells was significantly increased in Syn I KO compared to either WT or Syn II KO mice (Figure 4E). Notably, the percentage of BrdU/NeuN double-positive cells was also significantly augmented in Syn I KO animals indicating an increase in neuronal differentiation of newly generated cells that was accompanied by a parallel significant decrease in the percentage of differentiated glial cells (Figure 4F). The percentage of undifferentiated cells was similar in all genotypes (Figure 4F).

### Synapsin I and Synapsin II KO mice exhibit dysregulated BDNF signaling

**Figure 5:**
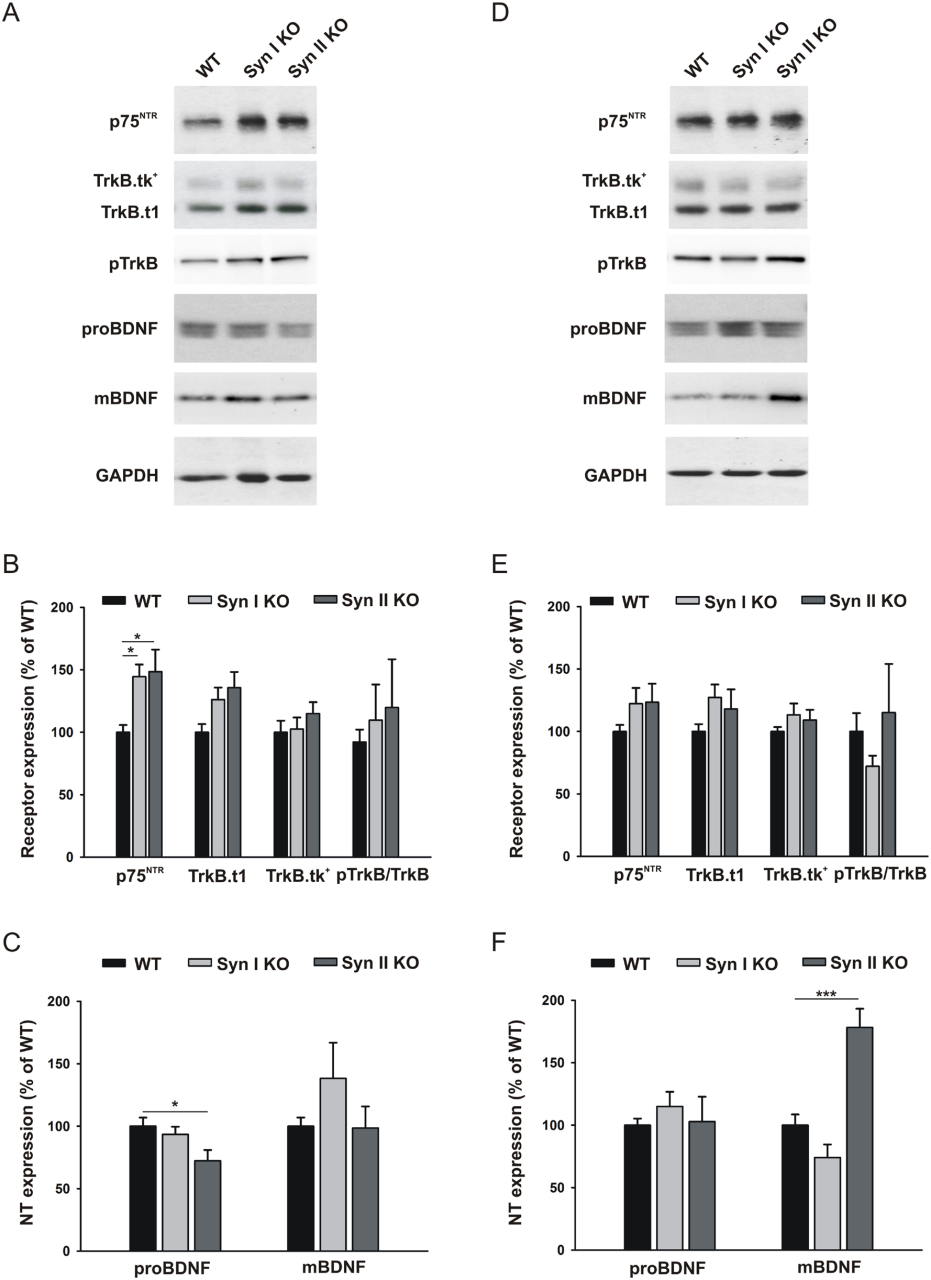
BDNF signaling in the DG of juvenile and symptomatic Syn I KO and Syn II KO mice **(A, D)** Representative immunoblots of the expression of p75^NTR^, full length TrkB receptor (TrkB.tk^+^), truncated TrkB (TrkB.t1), phosphorylated TrkB (pTrkB), proBDNF and mature BDNF (mBDNF) in the DG of 2-months-old (A) and 6-months-old (D) WT, Syn I KO and Syn II KO mice. Gapdh was used as control of equal loading. **(B, E)** Quantification of the expression levels of BDNF receptors and TrkB phosphorylation. **(C, F)** Quantification of the expression levels of proBDNF and mBDNF. Data (means ± sem) were normalized to Gapdh and expressed as percentages of the respective levels in WT mice (n = 4 for each genotype). ^*^ *p*<0.05, ^***^p<0.001, one-way ANOVA/Dunett’s test.

Neurotrophins and neurotrophin receptors have been implicated in the regulation of adult neurogenesis. BDNF signaling is a major candidate that can elicit distinct physiological effects via TrkB receptors that bind mature BDNF (mBDNF) and support neuronal growth, differentiation and survival, and p75^NTR^ that are activated by proBDNF, resulting in pro-apoptotic signaling [36]. Moreover, it is known that BDNF is one on the main activity-dependent determinants of the epileptogenic process [37] and that Syns are key effectors of BDNF signaling in neurons [38–40]. Thus, one possible mechanism by which Syn I and Syn II regulate adult neurogenesis is through an interplay with BDNF signaling. Thus, we analyzed the expression of a panel of regulatory molecules along the BDNF pathway in 2-months- and 6-months-old WT, Syn I KO and Syn II KO mice. Western blotting of DG extracts of 2-months-old mice revealed a significant increase of p75^NTR^ levels in both Syn I KO and Syn II KO with respect to WT mice (Figure 5A, 5B) and a decrease of proBDNF levels in Syn II KO mice (Figure 5A, 5C). On the other hand, at 6 months of age, a significant increase in mBDNF expression was only observed in Syn II KO DG in the absence of significant changes of BDNF receptors or proBDNF levels (Figure 5D–5F). These results suggest that the neurogenesis phenotype of Syn I KO and Syn II KO mice can be, at least in part, attributable to the dysregulation of neurotrophin signaling associated with the mutant genotype [40].

**Figure 6:**
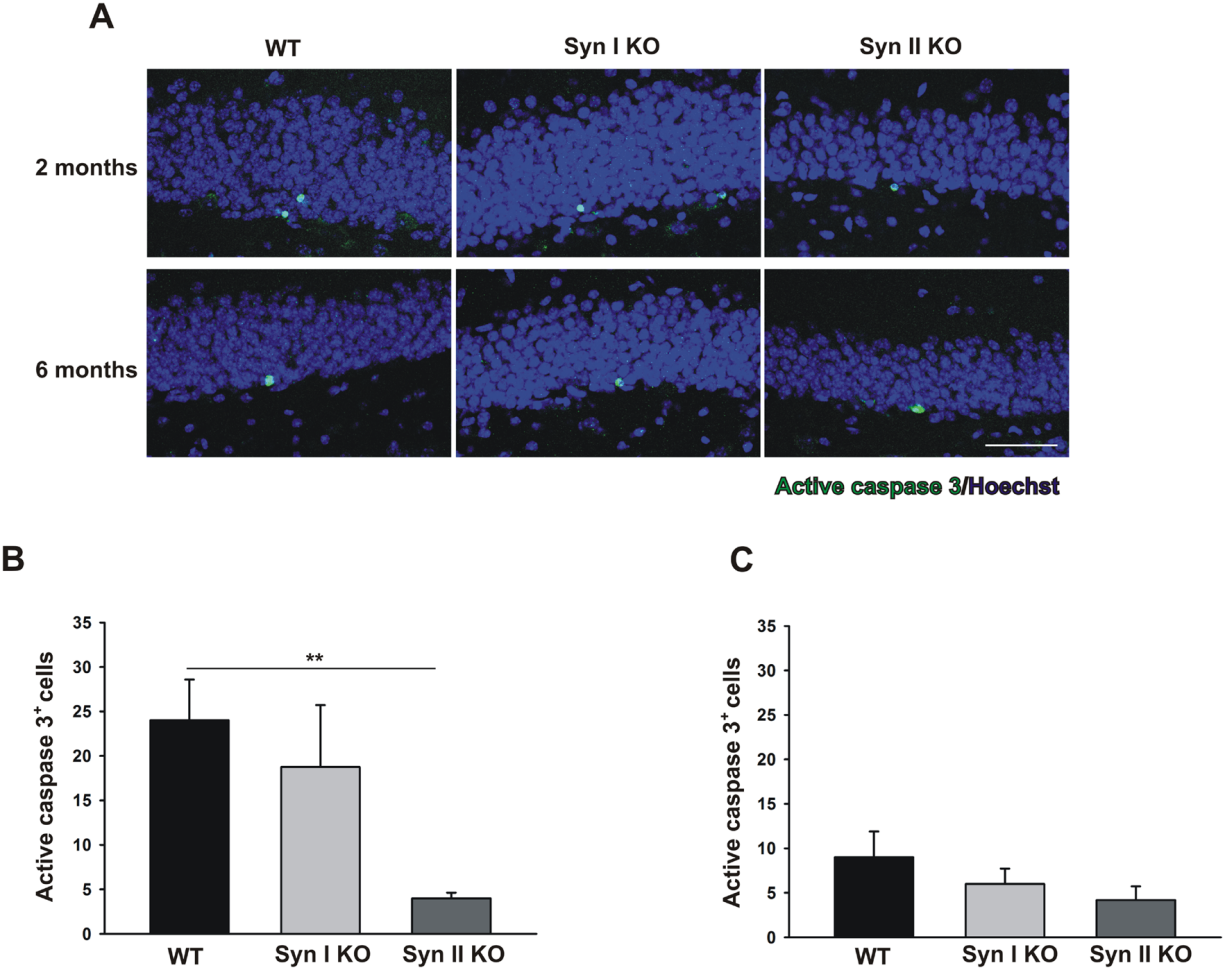
Apoptotic cell death rates in the DG of Syn I KO and Syn II KO mice **(A)** Immunofluorescence images of active caspase 3-labeled cells (green) in DG sections of 2- and 6-months-old WT, Syn I KO and Syn II KO mice. Sections were counterstained with Hoechst (blue) Scale bar, 50 μm. **(B, C)** Number of active caspase 3-positive cells in the subgranular zone of DG of 2-months-old WT (n=6), Syn I KO (n=4) and Syn II KO (n=7) mice (B) and 6-months-old WT (n=6), Syn I KO (n=6) and Syn II KO (n=5) mice (C). Data are presented as means ± sem. ^**^p<0.01, one-way ANOVA/Bonferroni’s test.

As neurotrophins are involved in the apoptotic pathways [36], we assessed whether the observed dysregulation of the BDNF signaling had an impact on cell death in Syn KO mice. Thus, we investigated active caspase 3-positive cells in 2- and 6-months-old WT and Syn KO animals. While the number of active caspase 3-positive cells was unaffected in Syn I KO mice at both ages with respect to the WT controls, Syn II KO mice showed a significant reduction of active caspase 3-positive cells at 2-months of age that became less pronounced at 6 months (Figure 6).

### Long-term potentiation in the DG of pre-symptomatic and symptomatic Syn I KO and Syn II KO mice

**Figure 7:**
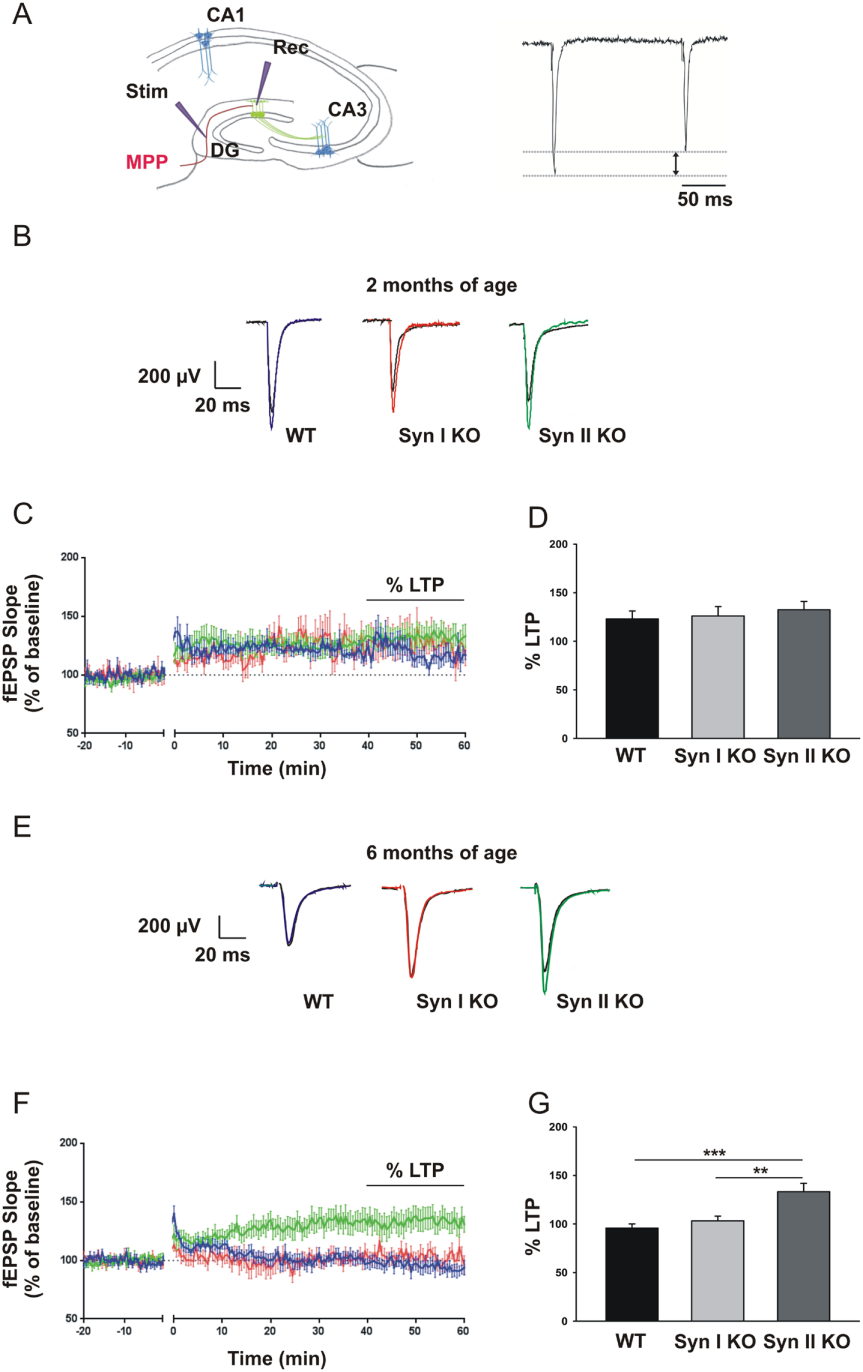
Neurogenesis-dependent LTP in the DG of juvenile and symptomatic Syn I KO and Syn II KO mice (**A)** Experimental configuration. *Left*: position of the stimulating and recording electrodes in the MPP and molecular layer, respectively. *Right*: the stimulation of the MPP fibers induces a PP depression. **(B, E)** Representative fEPSPs evoked in 2-months-old (B) and 6-months-old (E) WT, Syn I KO and Syn II KO hippocampal slices before (black traces) and after (colored traces) the induction of the LTP. **(C, F)** Plot of the fEPSP slope before and after the induction of the LTP (time 0) in the DG of 2-months-old (C) and 6-months-old (F) WT (blue traces), Syn I KO (red traces) and Syn II KO (green traces) mice. The extent of LTP was calculated as a percentage of the baseline between the last 40 and 60 min of recording. **(D, G)** Histogram of the LTP amplitude in 2-months-old (D) and 6-months-old (G) WT, Syn I KO and Syn II KO mice. Data are presented as mean ± sem. 2-months-old mice: n=8 slices from 4 WT; n=7 slices from 3 Syn I KO; n=7 from 3 Syn II KO. 6-months-old mice: n=10 slices from 4 WT; n=12 slices from 4 Syn I KO; n=12 from 4 Syn II KO. ^**^p<0.01, ^***^p<0.001, one-way ANOVA/Bonferroni’s test.

Next, we examined whether the changes in neurogenesis observed in Syn I KO and Syn II KO mice at pre-symptomatic and symptomatic temporal windows were associated with impaired DG synaptic plasticity. It has been demonstrated that, few weeks after becoming post-mitotic, DG newborn neurons exhibit increased excitability, enhanced synaptic plasticity and insensitivity to GABA inhibition. This condition causes a lower threshold for the induction of LTP that can be induced in the DG in the absence of GABA_A_ receptor blockade, which is necessary for inducing LTP at mature DG granule neurons [41–44]. Thus, extracellular recordings of field excitatory postsynaptic potentials (fEPSPs) in response to high-frequency stimulation (HFS) of the medial perforant path (MPP) were used to estimate the magnitude of newborn neuron-dependent LTP in the hippocampal DG in the absence of GABAergic inhibitors (Figure 7A).

When LTP was assessed in 2-months-old mice, no significant genotype-specific changes were observed. WT, Syn I KO and Syn II KO mice expressed a similar sustained potentiation of excitatory transmission of about 130% of the baseline (Figure 7B–7D). However, when newborn neuron-dependent LTP was assessed in 6-months-old symptomatic Syn I KO and Syn II KO mice and age-matched WT mice, LTP was practically absent in WT and Syn I KO animals, while it was preserved to approximately the same amplitude of the pre-symptomatic stage in Syn II KO mice (Figure 7E–7G).

## DISCUSSION

It is now widely accepted that the generation of new neurons is sustained throughout life thanks to the proliferation and differentiation of adult NPCs in specific regions of the mammalian brain. This process, referred as adult neurogenesis, is emerging as an important player in brain functions and homeostasis, while impaired or altered adult neurogenesis has been associated with a number of neuropsychiatric diseases, such as depression and epilepsy (reviewed by [45]). However, our understanding of the cellular and molecular mechanisms that regulate the neurogenic process in the adult brain, and how this may contribute to neurological alterations is still limited.

Here, we report that genetic deletion of either Syn I or Syn II alters distinct stages of neurogenesis in the hippocampal DG of juvenile mice. In particular, we observed a specific role for Syn II in the proliferation of neural progenitor cells, while Syn I appears to be mainly involved in the survival of the newborn neurons. As a result, the total number of newborn neurons was lower in both Syn I KO and Syn II KO mice, due to either decreased survival or proliferation rates, while the commitment of NPCs towards the neuronal phenotype was preserved. Since juvenile mice have not developed the epileptic syndrome yet, the observed effects can be directly related to the lack of expression of Syn I or Syn II or to secondary effects of their gene deletion.

Syns have been extensively described as essential modulators of proper neuronal development and function, such as establishment of neuronal polarity, neurite elongation and synapse formation, with non-redundant functions of the various isoforms [10, 11]. Syn III, the most precociously expressed Syn isoform, has been associated with profound alterations in adult neurogenesis, with decreased proliferation of NPCs and a compensatory increase in their survival and neuronal differentiation, resulting in no change in the number of newborn neurons and in the DG volume [32]. The alterations of adult neurogenesis in Syn I KO and Syn II KO mice testifies that, although the expression of both Syn I and Syn II is boosted in developing neurons and peaks at the time of synapse formation, the proteins play a role also in the more precocious stages of neurogenesis. Indeed, a previous report described a decrease in the number of Ki67- and DCX-positive cells in 2-months-old Syn II KO [46] consistently with our data in the pre-symptomatic stage of these mice.

The distinct neurogenic phenotype of Syn I KO and Syn II KO mice in the proliferation and survival of NPCs correlates with the distinct developmental roles and biochemical properties of the two Syn isoforms [10, 11]. In spite of the different mechanism, however, the number of surviving NPCs is decreased by approximately the same extent in the absence of either isoform.

It is also possible that the perturbation of the neurogenesis, characterizing Syn I KO and Syn II KO mice, is attributable, at least in part, to secondary effects of gene deletion. We recently demonstrated that primary cortical neurons of both Syn I KO and Syn I/II/III KO display increased levels of p75^NTR^ [40], a result that we confirmed here *in vivo* for both mutant genotypes at 2 months of age. Thus, the decreased neuronal survival rate of Syn I KO may be due to the increased p75^NTR^ signaling. By contrast, the concomitant decrease of the p75^NTR^ ligand proBDNF in Syn II KO mice could dampen the effects of increased p75^NTR^ signaling, resulting in unaltered or even increased survival rate of neural progenitors. Indeed, the number of apoptotic cells was significantly reduced in 2-months-old Syn II KO mice. However, the reduced number of apoptotic cells in 2-months-old Syn II KO mice can also be contributed by the reduced proliferation rate observed in these mice. In 6-months-old mice, both proliferation and apoptotic rates were lower than in younger animals. At this age, Syn II KO mice showed the higher proliferation rate, but also a trend towards lower apoptotic elimination. This apparent discrepancy can be, at least in part, attributable to the concomitant sharp increase in mBDNF in 6-months-old Syn II KO mice that may downregulate the apoptotic process.

In mice lacking Syn I or Syn II, epileptic seizures start developing approximately at 2-3 months of age, progressively aggravate with age [22]. Thus, any alteration of neurogenesis in symptomatic Syn I KO and Syn II KO mice may result from both the genotype and the appearance of epileptic seizures. Seizure activity involving the hippocampus strongly affects adult neurogenesis in the DG, although it is not clear whether seizure-induced aberrant neurogenesis is part of the epileptogenic process and contributes to the disease or represents an attempt to repair the injured brain structures and compensate for the neuronal loss due to seizures [47–49]. Further studies will be needed to understand weather changes in neurogenesis are a consequence of epilepsy in Syns KO mice or if instead changes in neurogenesis play a role in the establishing the epileptic phenotype in these mice. Nevertheless, in epileptic Syn I KO and Syn II KO mice, the neurogenic defects observed in the pre-symptomatic phase were compensated, with a resulting increase in NPC proliferation in Syn II KO and of NPC survival in Syn I KO mice. The effect on NPC proliferation and the increase in mBDNF expression were particularly intense in Syn II KO mice, in parallel with the higher severity of seizure activity in these mice. BDNF is one of the main actors of epileptogenesis and of epilepsy-induced neurogenesis. Indeed, it has been shown that intra-hippocampal infusion of BDNF in adult rats increases DG neurogenesis, while heterozygous BDNF KO mice display impaired NPC proliferation [50, 51].

It is known that manipulations that inhibit adult neurogenesis prevent the induction of LTP evoked by MPP stimulation in the DG of slices with intact GABAergic transmission [41, 43]. This demonstrates that this form of long-term plasticity is mediated by newborn granule neurons that are subjected to a lower inhibitory tone than mature DG granule cells. In spite of the changes in NPC survival and proliferation observed in juvenile Syn I KO and Syn II KO mice, respectively, no major genotype-dependent effects in the expression of LTP were observed. This indicates that, notwithstanding the alterations in NPC generation and survival observed in pre-symptomatic Syn KO mice, the final output of post-mitotic DG newborn neurons is sufficient to express physiological amplitudes of newborn neuron-dependent DG-LTP. At 6 months of age, no newborn neuron-dependent LTP was elicited in WT mice, consistent with the previously reported decrease of threshold and amplitude of newborn neuron-dependent LTP with aging [52, 53]. In spite of the similar epileptic phenotype, LTP was maintained in Syn II KO mice, but was absent in Syn I KO mice. The specific preservation of LTP in Syn II KO may result from several factors, including: (i) the observed increase in NPC proliferation and increased mBDNF levels in symptomatic Syn II KO mice and (ii) the distinct epileptogenic pathways recently identified in Syn I and II KO mice. Although both Syn I KO and II KO mice display dysfunctions in GABAergic transmission resulting in an excitation/inhibition imbalance, deletion of Syn I generates a deficit of phasic inhibition [26, 27], while deletion of Syn II abolishes tonic inhibition [29, 30]. Recent experimental evidence has demonstrated that increased tonic inhibition induced by treatment with agonists of extrasynaptic GABA_A_ receptors [54] or by altered astrocyte GABA release [55] impaired LTP in the hippocampal DG. Thus, the lack of GABAergic tonic inhibition that we identified in DG granule cells of the Syn II KO could explain the persistence of LTP that characterizes the DG of symptomatic Syn II KO mice.

In conclusion, our findings implicate Syn I and Syn II in distinct aspects of adult DG neurogenesis, providing a basis for further explorations of the molecular mechanisms underlying the modulation of neurogenesis in the adult brain. Since deficiency or mutations of Syn I and Syn II are associated with the emergence of epilepsy and autism spectrum disorders in humans, our results open interesting questions on the role of neurogenesis in the pathogenesis of these diseases. Further studies are needed to clarify the interplay between the neurogenesis deficits and the brain alterations underlying the appearance of the epileptic/autistic phenotype in these mice.

## MATERIALS AND METHODS

### Histological evaluation of neurogenesis

Neurogenesis quantification was performed on 2- and 6-months-old C57BL/6J WT, Syn I KO and Syn II KO male mice and WT littermates using the thymidine analog BrdU [45, 56]. All animal experiments were performed in accordance with the guidelines established by the European Community Council (Directive 2010/63/EU of September 22, 2010) and were approved by the Italian Ministry of Health. The animals were injected intraperitoneally with 100 mg/Kg body weight BrdU once daily for 6 days. One day and 28 days after the last BrdU injection, animals were deeply anesthetized and transcardially perfused with 4 % paraformaldehyde in 100 mM phosphate buffer, pH 7.4. Coronal sections (30 μm) were cut on a freezing sliding microtome (Thermo Scientific HM 450). For BrdU immunohistochemistry, sections were pretreated with 2 N HCl as previously described [8] to denature DNA, washed with phosphate-buffered saline (PBS) and incubated for 1 h at room temperature in PBS containing 0.1 % Triton (PBST) and 5 % fetal bovine serum (blocking buffer). Sections were then incubated overnight at 4°C with the following primary antibodies in blocking buffer: rat anti-BrdU (Abcam 1:200), mouse anti-NeuN (Millipore 1:250), rabbit anti-DCX (Abcam 1:1000), rabbit anti-GFAP (Abcam 1:200) and rabbit anti-active caspase 3 (R&D System 1:500). After washing in PBST, secondary antibodies Alexa Fluor 488 goat anti-rat IgG, Alexa Fluor 568 goat anti-rabbit IgG, Alexa Fluor 647 goat anti-mouse IgG (Molecular Probes) 1:1000 were incubated for 2 h at room temperature. Sections were counterstained with Hoechst 33342 (Sigma-Aldrich) to detect nuclei, mounted with Prolong Gold (Invitrogen), and coverslipped. Images were taken with a Nikon A1 confocal microscope through a 40x objective.

### Quantification of BrdU-, BrdU/NeuN-, BrdU/DCX- and active caspase 3-positive cells

For the assessment of adult DG neurogenesis and active caspase 3-positive cells, stereological cell counting was performed in serial coronal sections (180 μm pitch) covering the complete rostro-caudal extension of the DG, as previously described [8]. Fluorescence images were captured with a Nikon A1 confocal scanning microscope equipped with a 40x air objective and a motorized stage. For each section, confocal z-stack images (1 μm z-step size) covering the complete DG were acquired and DG reconstructed with the NIS Element AR software (Nikon). Immunopositive cells in the granular cell layer (GCL) and the subgranular zone (SGZ, defined as a 10 μm region below the GCL) were counted on the reconstructed images by an operator blind to the experimental groups according, to the optical dissector principle [57].

### Assessment of DG and hilus volume

For the quantification of DG and hilus volumes, slices were stained with NeuN and acquired by confocal microscopy. The profile area of each section was measured using NIS-Elements AR Microscope Imaging Software. The volume estimation of these brain regions was obtained by multiplying the sum of the areas of all sections by the distance between the sections applying the principle of Cavalieri [58].

### Western blotting analysis

Mice were euthanized, brains removed and hippocampi dissected out, quickly frozen and stored at −80°C. Frozen tissues were homogenized at 4°C in 200 μl of 1X cold PBS containing a cocktail of protease and phosphatase inhibitors (Halt Phosphatase Inhibitor Cocktail, Thermo Scientific) and sonicated two times for 10 s. Samples were then clarified by centrifugation at 1000 x g for 10 min at 4°C and the supernatant recovered. Protein concentrations were determined using the colorimetric Bradford assay (Bio-Rad Laboratories). Equivalent amounts of proteins (50-150 μg/lane) were subjected to sodium dodecyl sulfate–polyacrylamide gel electrophoresis (SDS–PAGE) on 9 % polyacrylamide gels and blotted onto nitrocellulose membranes (Whatman, Sigma-Aldrich). After the transfer, membranes were blocked with 5 % non-fat dry milk or BSA in Tris-buffered saline (TBS) containing 0.1 % Triton X-100 (TBST) for 1 h at room temperature and incubated either overnight at 4°C or for 2 h at room temperature with the following primary antibodies: rabbit anti-GAPDH (1:5000; Abcam), rabbit anti-p75^NTR^ (1:1000; Cell Signaling), rabbit anti-BDNF and ^Y706^P-TrkB (1:500; Santa Cruz Biotechnology). After three washes, membranes were incubated for 1 h at room temperature with horseradish peroxidase (HRP)-conjugated anti-rabbit (1:3000; Bio-Rad Laboratories) antibodies. Bands were revealed with the ECL chemiluminescence detection system (Thermo Scientific) and fluorograms were quantified by densitometric analysis.

### Hippocampal slices and field recordings

Horizontal hippocampal slices were obtained from 2- and 6-months-old WT, Syn I and II KO male littermates. Mice were anaesthetized with isoflurane and decapitated; the brain was quickly removed and immersed in an ice-cold ‘cutting’ solution saturated with 95% O_2_ and 5% CO_2_ and composed of (mM): 87 NaCl, 25 NaHCO_3_, 2.5 KCl, 0.5 CaCl_2_, 7 MgCl_2_, 25 glucose, 75 sucrose. The dissected hemispheres were cut into 400 μm thick slices using an HM 650 Vibratome (Microm International GmbH, Walldorf, Germany). Slices were incubated at 35°C for 1 h and then stored at room temperature in ‘standard recording solution’ saturated with 95 % O_2_ and 5 % CO_2_ and composed of (mM): 125 NaCl, 25 NaHCO_3_, 25 glucose, 2.5 KCl, 1.25 NaH_2_PO_4_, 2 CaCl_2_, 1 MgCl_2_. Slices were transferred into a ‘submerged’ recording chamber continuously superfused at a rate of 1.5 ml/min with the ‘standard recording solution’. Synaptic inhibitors were not used in order to induce a neurogenesis-dependent LTP as previously described [41]. The bath temperature was monitored and maintained at 30 ± 1°C. Extracellular field potentials were recorded in the molecular layer of the DG with glass microelectrodes (1-2 MOhm) filled with standard recording solution. Electrical stimulation was delivered through a monopolar glass electrode connected with an isolated pulse stimulator (A-M Systems, Carlsborg, WA) positioned into the MPP. We applied paired-pulse (PP) protocol at 200 ms to test the correct position of the stimulation electrode, since the MPP-DG granule cell synapse is characterized by PP depression [59]. The stimulation intensity was determined based on the input/output (I/O) relationship, and the stimulation intensity that produced 50% of the maximal response was used for test pulses and tetanic stimulation. The stimulus duration was 0.1 ms. Field EPSPs were acquired by using a MultiClamp 700B (Axon Instruments, Molecular Devices, Sunnyvale, CA, USA) amplifier and the pClamp 9.2 software (Axon Instruments). The fEPSP slope was evaluated as a linear regression of the rise phase from 20% to 80% of the fEPSP amplitude using Clampex 9.2 software (Axon Instruments). For each experiment, the ‘baseline slope’ was calculated by averaging the slope values obtained after the stimulation of the MPP fibers at 0.03 Hz for 20 min. The magnitude of fEPSPs was measured as slope normalized to baseline and plotted against time. To study LTP, baseline responses were evoked every 30 s for 20 min before LTP induction. LTP was evoked by 4 trains at 100 Hz, 500 ms each, repeated every 20 s. Time-point 0 marks the application of the first LTP-inducing stimulus. The post-tetanic responses were then measured every 30 s for 60 min. LTP was estimated as percentage change of the baseline fEPSP slope and, for each recording, the final magnitude of LTP was determined as the average of responses between 40 and 60 min post-induction.

### Statistical analysis

Data are reported as means ± sem throughout. Normal distribution of data was assessed using the D’Agostino-Pearson’s normality test. When data were normally distributed, one- or two-way ANOVA followed by either Dunnett’s or Bonferroni’s multiple comparison test. The significance level was preset to p<0.05. Statistical analysis was carried out using Prism/GraphPad (GraphPad Software, Inc.) software.

## Abbreviations

ASD: autism spectrum disorder
BDNF: brain-derived neurotrophic factor
BrdU: 5-bromo-2′-deoxyuridine
DCX: doublecortin
DG: dentate gyrus
fEPSPs: field excitatory postsynaptic potentials
GCL: granular cell layer
GFAP: glial fibrillary acidic protein
HFS: high-frequency stimulation
KO: knockout
LTP: long-term potentiation
MPP: medial perforant path
NPCs: neural progenitor cells
PP: paired-pulsed
SGZ: subgranular zone
SV: synaptic vesicle
SYNS: synapsins
WT: wild type
